# Association between intrahepatic cccDNA and the severity of liver inflammation in chronic hepatitis B virus infection patients

**DOI:** 10.3389/fmed.2024.1519686

**Published:** 2025-01-24

**Authors:** Suxian Zhao, Chen Dong, Chudi Chang, Jiaming Zhang, Jing Li, Xiaoxiao Zhang, Weiguang Ren, Ying Zhang, Yuemin Nan

**Affiliations:** Department of Traditional and Western Medical Hepatology, Hebei Medical University Third Hospital, the Key Laboratory of Hepatic Fibrosis Mechanisms of Chronic Liver Diseases in Hebei Province, Hebei International Science and Technology Cooperation Base – Hebei International Joint Research Center for Molecular Diagnosis of Liver Cancer, Shijiazhuang, China

**Keywords:** covalently closed circular DNA (cccDNA), inflammation, liver, HBV, chronic hepatitis B

## Abstract

**Background and aims:**

This research aimed to examine the association between hepatitis B virus (HBV) covalently closed circular DNA (cccDNA) and liver inflammation in chronic hepatitis B (CHB) infection patients.

**Methods:**

From August 2013 to June 2022, CHB patients at Hebei Medical University Third Hospital (Hebei, China) were recruited. Intrahepatic cccDNA was quantified and its association with liver inflammation was analyzed. Liver inflammation was assessed using the Ishak-modified histological activity index (HAI). Biochemical and viral indicators as well as hepatic inflammation biomarkers were monitored.

**Results:**

In total, 55 CHB patients were enrolled. The average HBV-cccDNA level was markedly elevated in HBeAg+ patients compared to HBeAg patients. Intrahepatic cccDNA levels differed significantly in different liver inflammation groups and showed a positive correlation with the HAI score for hepatic inflammation.

**Conclusion:**

HBV-cccDNA level was associated with liver inflammation.

## 1 Introduction

Hepatitis B virus (HBV) is a significant contributor to viral hepatitis, which affects over 257–291 million individuals globally. The interplay between HBV viral propagation and the immune system’s reaction gives rise to various health consequences, from liver inflammation to hepatocellular carcinoma (HCC) and cirrhosis (1). HBV-cccDNA formation is critical for the virus’s life cycle and is crucial in the persistence and recurrence of infection. Hence, intrahepatic HBV-cccDNA acts as an essential biomarker of disease progression and is widely utilized to assess the effectiveness of antiviral treatments and establish treatment goals (2–6).

The host anti-HBV-specific immune response can control infection by clearing infected hepatocytes. However, the virus can weaken and deplete the host’s specific antiviral cellular immune system by continuously expressing high levels of hepatitis-B-e-antigen (HBeAg) and hepatitis-B-surface-antigen (HBsAg), making it ineffective in clearing infected hepatocytes. In clinical practice, the negative conversion of HBeAg commonly signifies partial immune control over chronic hepatitis B (CHB) infection. Therefore, patients are assigned to HBeAg+ chronic HBV group, HBeAg+ CHB group, HBeAg chronic HBV infection group, and HBeAg CHB group according to their HBeAg status and severity of liver inflammation. The progression of persistent HBV relies on the interplay between the virus and host immunity. However, due to the lack of simple and effective host-specific immune evaluation indicators for HBV, the dynamic changes of host immune factors in persistent HBV and their role in disease progression remain largely unknown. Previous studies have shown that host immune activation against HBV can effectively suppress viral replication by targeting infected liver cells, but this response is typically seen in the early HBV stage in HBeAg+ patients with high levels HBV-DNA levels (7). HBV can undergo spontaneous reactivation or reactivate in response to immunosuppressive or anti-inflammatory treatments (8). The existing nucleos(t)ide analogs (NAs) inhibit the replication of cytoplasmic HBV; however, they do not address cccDNA and are not curative treatments (9). Magri et al. (10) revealed a gene signature related to immune responses involved in HBV-cccDNA transcription. In addition to aspartate transaminase (AST) and ALT, serum HBsAg level was found to correlate with the grade of inflammation in HBeAg+ CHB patients prior to NA treatment. Additionally, combining HBsAg with AST demonstrated outstanding diagnostic performance for predicting severe inflammation (11).

Therefore, this research aimed to evaluate the changes in HBV-cccDNA levels of CHB patients receiving liver biopsy based on their HBeAg status, liver function, liver tissue inflammation activity, viral load, and cccDNA levels.

## 2 Materials and methods

### 2.1 Objects

From August 2013 to June 2022, 55 patients with CHB at Hebei Medical University Third Hospital (Hebei, China) were recruited in this study. Persistent HBV patients receiving liver biopsy and screening for serum biochemical and viral indicators were also included. The following criteria were used for inclusion: (1) age 18–65 years; (2) the serum level of HBsAg was detected in the last 6 months; (3) the patient provided consent for a liver biopsy and testing of serum biochemical and viral indicators; and (4) informed consent in writing was provided for treatment and participation in this research. The following criteria were used for exclusion: (1) the patient had previously undergone antiviral therapy for HBV; (2) co-infection with other viruses, including HCV, HDV, and HIV; (3) diagnosed with compensated/decompensated cirrhosis or HCC; (4) autoimmune hepatitis, primary biliary cirrhosis, alcoholic/non-alcoholic liver diseases; and (5) coexisting conditions, or severe metabolic imbalance or psychiatric disorders. This research was granted approval from the Ethics Committee of the same institute.

### 2.2 Data collection

Demographic and laboratory parameters including age, gender, red blood cell (RBC) count, white blood cell (WBC) count, hemoglobin (HGB) level, platelet (PLT) count, and the levels of alanine aminotransferase (ALT), AST, alkaline phosphatase (ALP), albumin (ALB), total bilirubin (TBIL), glutamyl transpeptidase (GGT), HBsAb, HBsAg, HBcAb, HBeAg, HBeAb, and HBV-DNA were obtained retrospectively from the electronic medical records system.

### 2.3 Assessment of liver inflammation

Liver biopsies were conducted through standard procedures. Ultrasound-guided liver biopsy was conducted based on the standard procedure (12). At least 2 pieces with 2.0 cm length were collected to ensure the presence of 11 portal tracts for histological evaluation. Two pathologists independently evaluated the biopsy specimens, blinded to both the biopsy timing and clinical information. In cases of inconsistency, both pathologists reexamined the samples to reach a consensus. Liver inflammation was assessed using the Ishak-modified histological activity index (HAI) (13, 14).

### 2.4 Detection of intrahepatic HBV DNA and cccDNA levels

About 30 μm formalin fixed paraffin-embedded liver biopsy tissue in sections of 6 μm each was used for DNA extraction. To prevent contamination, disposable tweezers, brush, and interleaver were used and sectioning blades were carefully cleaned with 70% ethanol after every sampling. Genomic DNA and intracellular-free microchromosomal DNA were isolated using the QIAamp-DNA-Mini-Kit in accordance with the manufacturer’s guidelines (QIAGEN, Germany). PSAD was utilized for digestion of HBV double-stranded DNA, relaxed circular DNA, and single-stranded DNA (Epicentre, USA). Subsequently, cccDNA-selective amplification was carried out using the rolling-circle-amplification (RCA) technique with Phi29 (New England Biolabs, USA). RCA products were further amplified and quantified via TaqMan real-time PCR, employing probes that target the unfilled portions of the viral genome along with cccDNA-specific primer pairs. Notably, Phi29 and PSAD were unnecessary for detecting total HBV-DNA. The cell count was determined using probes and primers as a standard control, specifically DNA fragments of human β-actin. The LLOQ was 0.01 copies per cell.

### 2.5 Statistical analysis

All statistical tests were conducted using GraphPad Prism v8.0, MedCalc v15.0, and SPSS v26.0. Continuous data are presented as mean ± SD or median (IQR). They were compared using the Kruskal–Wallis test. Categorical data were examined using the χ^2^ test. They are presented as numbers (percentages). The correlations between two parameters were determined using Pearson’s correlation. Two-tailed tests of significance were conducted, and *p* < 0.05 was regarded as statistically significant.

## 3 Results

### 3.1 Baseline features

In total, 55 patients were included between August 2013 and June 2022. These patients were categorized into two groups according to their HBeAg status. No remarkable differences were observed between the two groups regarding sex, age, AST, total bilirubin, and albumin between the HBeAg and HBeAg+ groups. However, obvious differences were noted between the two groups regarding AST, GGT, serum HBV-DNA, serum HBsAg, intrahepatic HBV-DNA, and intrahepatic HBV-cccDNA levels (Table 1).

**TABLE 1:** Baseline characteristics.

	HBeAg-negative patients (*n*=26)	HBeAg-negative patients (*n*=29)	*t*	*p*
Sex (male/female)	21/7	17/10	0.933	0.334
Age (years)	37.11 ± 12.73	39.81 ± 10.46	−1.121	0.262
WBC (10^9^/L)	5.45 ± 1.37	6.02 ± 1.35	−1.704	0.088
HGB (g/L)	154.00 (136.00, 162.00)	152.90 (129.60, 160.00)	−0.459	0.647
PLT (10^9^/L)	189.46 ± 70.90	209.28 ± 58.20	−1.107	0.268
Albumin (g/L)	46.45 (40.48, 48.68)	47.40 (44.15, 4872)	−1.136	0.256
ALT (U/L)	51.50 (29.00, 131.75)	27.00 (22.00, 50.00)	−2.467	0.014
AST (U/L)	32.00 (21.40, 71.50)	25.00 (20.00, 30.00)	−1.912	0.056
Total bilirubin (μmol/L)	17.52 (12.64, 22.94)	14.28 (12.06, 22.35)	−0.892	0.372
ALP (U/L)	69.50 (52.42, 88.00)	70.00 (48.00, 90.00)	−0.044	0.965
GGT (U/L)	43.50 (27.50, 68.50)	25.00 (16.00, 44.00)	−2.90	0.022
HBsAg (log10 IU/mL)	3.89 (3.51, 4.50)	2.97 (1.97, 3.72)	−3.862	0.000
HBeAg (log10 IU/mL)	2.43 (1.15, 3.06)	−0.40 (−0.49, −0.37)	−6.186	0.000
Serum HBV-DNA (log10 IU/mL)	7.16 (4.71, 8.00)	3.14 (2.09, 3.94)	−4.694	0.000
Intrahepatic HBV-DNA (log10 IU/mL)	1.83 (0.99, 2.83)	−2.00 (−2.00, 0.52)	−4.306	0.000
Intrahepatic cccDNA (copies/cell)	0.53 (0.04, 3.25)	0.01 (0.01, 0.05)	−3.587	0.000

HBeAg, hepatitis B e antigen; WBC, white blood cell; RBC, red blood cell, HGB, hemoglobin; PLT, platelet; ALT, alanine aminotransferase; AST, aspartate aminotransferase; ALP, alkaline phosphatase; GGT, glutamyl transpeptidase; HBV, hepatitis B virus; HBsAg, hepatitis B surface antigen; cccDNA, covalently closed circular DNA.

### 3.2 Variation in HBV-cccDNA and serum-based viral indicators

**FIGURE 1:**
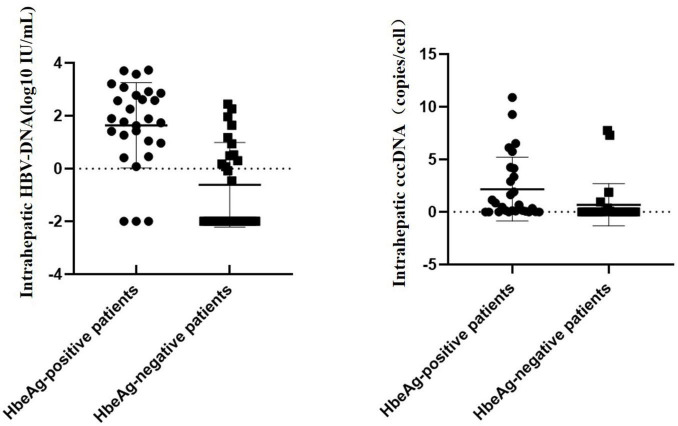
Hepatitis B virus-DNA and cccDNA levels in HBeAg+ and HBeAg patients.

When stratifying patients by the HBeAg status, the mean HBV-cccDNA and intrahepatic HBV-DNA levels of patients with HBeAg+ were remarkably increased compared to those with HBeAg (0.53 vs. 0.01 log10 copies/cell, 1.83 vs. 2 log10 IU/mL, *p* < 0.001) (Figure 1).

### 3.3 HBV-cccDNA and hepatic inflammation

**FIGURE 2:**
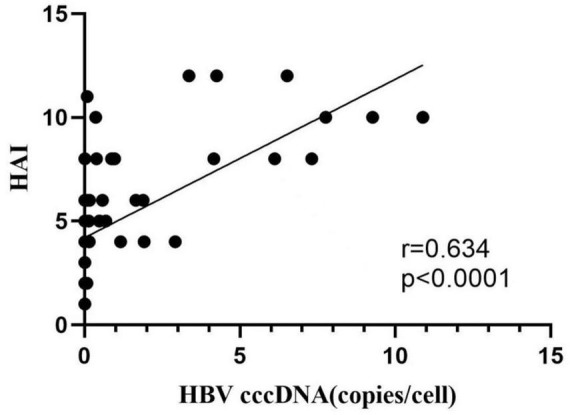
Correlation between HBV cccDNA and HAI.

Fifty-five patients with ALT < 2 ULN were categorized into two groups according to the severity of hepatic inflammation. Twenty-one patients with liver inflammation (ALT more than 2 ULN) were allocated into group A. Thirty-four patients without liver inflammation (ALT less than 2 ULN) were allocated into group B. Age, sex, and ALB did not markedly vary between the two groups. There were obvious differences in AST, ALT, GGT, total bilirubin, serum HBsAg, serum HBeAg, intrahepatic HBV-DNA levels, serum HBV-DNA, and intrahepatic HBV-cccDNA levels between the two groups (Table 2). Besides, logistic regression analysis revealed positive correlations between HBV-cccDNA levels and liver inflammation HAI scores (Figure 2).

**TABLE 2:** Expression levels of cccDNA in different inflammation groups.

	Group A (*n*=21)	Group B (*n*=34)	*t*	*p*
Sex (male/female)	17/4	21/13	2.238	0.135
Age (years)	39.00 (31.00, 45.50)	37.00 (30.75, 42.00)	−0.217	0.828
WBC (10^9^/L)	5.67 ± 1.37	5.76 ± 1.41	−0.257	0.797
HGB (g/L)	152.00 (132.50, 156.50)	154.00 (134.75, 161.80)	−0.639	0.523
PLT (10^9^/L)	206.20 ± 67.67	187.70 ± 60.87	−1.100	0.271
Albumin (g/L)	46.94 ± 3.52	42.85 ± 6.82	−1.784	0.074
ALT (U/L)	92.00 (42.50, 187.50)	28.00 (22.75, 46.00)	−4.177	0.000
AST (U/L)	54.00 (32.00, 85.0)	22.50 (20.00, 27.00)	−4.430	0.000
Total bilirubin (μmol/L)	22.53 (13.79, 25.78)	14.03 (12.02, 20.00)	−2.460	0.014
ALP (U/L)	68.00 (48.00, 104.00)	71.00 (52.43, 88.50)	−0.250	0.802
GGT (U/L)	59.00 (35.00, 83.50)	23.50 (16.00, 37.75)	−4.150	0.000
HBsAg (log10 IU/mL)	3.89 (3.18, 4.21)	3.49 (2.48, 3.84)	−1.625	0.104
HBeAg (log10 IU/mL)	2.36 (−0.36, 3.04)	−0.36 (−0.43, 1.14)	−2.461	0.014
Serum HBV-DNA (log10 IU/mL)	6.58 (3.39, 7.97)	3.68 (2.42, 5.16)	−2.384	0.017
Intrahepatic HBV-DNA (log10 IU/mL)	1.64 (0.46, 2.59)	0.13 (−2.00, 1.91)	−2.181	0.029
Intrahepatic cccDNA (copies/cell)	0.69 (0.20, 5.19)	0.01 (0.01, 0.41)	−2.967	0.003

HBeAg, hepatitis B e antigen; WBC, white blood cell; RBC, red blood cell, HGB, hemoglobin; PLT, platelet;ALT, alanine aminotransferase; AST, aspartate aminotransferase; ALP, alkaline phosphatase; GGT, glutamyl transpeptidase; HBV, hepatitis B virus; HBsAg, hepatitis B surface antigen; cccDNA, covalently closed circular DNA. Group A: patients with inflammation (serum ALT level above 2ULN); group B: patients who did not experience inflammation (serum ALT level below 2ULN).

### 3.4 Correlations between HBV-cccDNA and viral indicators

**FIGURE 3:**
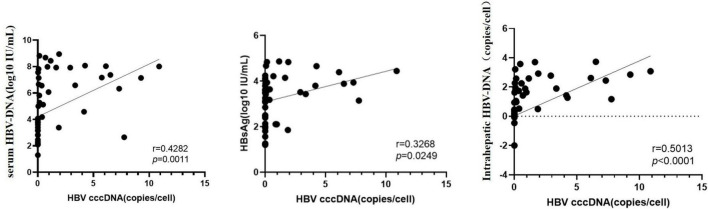
Correlation between HBV cccDNA and virological markers.

Pearson correlation analysis indicated that intrahepatic cccDNA level was positively correlated with HBsAg, HBeAg, HBeAb, and serum HBV-DNA levels (*r* = 0.312, 0.314, 0.295, 0.403, *p* < 0.05). Intrahepatic HBV-DNA level was positively correlated with HBsAg, HBeAg, HBeAb, serum HBV-DNA, and intrahepatic HBV-cccDNA levels (*r* = 0.618, 0.700, 0.625, 0.815, and 0.501, *p* < 0.001) (Figure 3 and Table 3).

**TABLE 3:** Correlation between HBV-DNA, cccDNA and liver function, virological markers.

	Intrahepatic HBV-DNA (copies/cell)		Intrahepatic cccDNA (copies/cell)	
	***r***	***p***	***r***	***p***
ALT (U/L)	−0.109	0.428	0.001	0.996
AST (U/L)	−0.138	0.323	−0.007	0.962
HBsAg (log10 IU/mL)	0.618	<0.001	0.312	0.033
HBeAg (log10 IU/mL)	0.700	<0.001	0.314	0.030
HBeAb (log10 IU/mL)	0.625	<0.001	0.295	0.042
HBcAg (log10 IU/mL)	0.136	0.355	0.103	0.486
serum HBV-DNA (log10 IU/mL)	0.815	<0.001	0.403	0.002
Intrahepatic cccDNA (copies/cell)	0.501	<0.001	—	—

## 4 Discussion

Our findings indicated that the HBV-cccDNA level was markedly higher in HBeAg+ patients than in HBeAg patients. The reduced viral load in serum during the HBeAg phase seems to be associated with mechanisms other than merely the repression of intracellular HBV-cccDNA, and the replicative effectiveness of HBV is also involved in this phenomenon (15). Furthermore, the reduced levels of intracellular pregenomic RNA also indicate the suppression of serum HBV-DNA in HBeAg patients. Additionally, the serum HBV-DNA concentration is affected by the amount of intracellular HBV-cccDNA and the virus’s replication efficiency (16).

Our results indicated that serum viral markers positively related to intrahepatic HBV-cccDNA levels in HBsAg, HBeAg, HBeAb, and HBV-DNA, which are in agreement with prior research (17–19). For instance, Thompson et al. (18) found that numerous hepatocytes stained positive for HBsAg in HBeAg+ patients with suppressed viral propagation, suggesting that the link between HBV replication and HBsAg production weakens during the HBeAg stage. This is likely due to HBsAg being produced from sources other than intracellular HBV-cccDNA. Additionally, prior research has reported a correlation between serum HBsAg levels and cccDNA copy number (20).

In our study, intrahepatic HBV-cccDNA levels, along with HBV-DNA viral load, HBeAg, and HBsAg titers, were substantially higher in HBeAg+ patients than in HBeAg patients, likely due to increased viral protein synthesis and release in individuals with elevated intracellular HBV-cccDNA levels. Such immune activation may worsen inflammation, resulting in elevated serum ALT concentrations. Intrahepatic HBV-cccDNA level was evaluated through liver biopsies and histological assessment. This is usually accompanied by hepatocyte damage, hepatic flares, and histologic changes. HBV-cccDNA transcribes mRNA encoding HBV-specific proteins and can recruit and contribute to inflammatory cells that cause liver injury (21). The findings indicated that elevated intrahepatic HBV-cccDNA level could elevate the likelihood of hepatic inflammation. More research is needed to confirm HBV-cccDNA as a viral indicator for patients potentially requiring further outpatient support. Currently, there is controversy regarding the association between intrahepatic HBV-cccDNA and histopathological liver inflammation (22–25). Some investigators have found no significant association between HBV-cccDNA levels and hepatic inflammation grade in HBeAg patients, whereas other groups report that HBeAg patients with significant hepatic inflammation show elevated intrahepatic HBV-cccDNA levels. Magri et al. (10) showed that transcripts derived from cccDNA are linked to biomarkers of hepatic inflammation. The inflammatory hepatic transcriptome analysis indicated that 24 genes are remarkably correlated with cccDNA transcriptional activity (10). The study identified a gene signature related to the immune response associated with HBV-cccDNA transcription.

Herein, we prospectively examined the association between intracellular HBV-cccDNA content and hepatic inflammation in patients. HBV-cccDNA can be used as a biomarker for assessing liver disease severity in clinical practice. However, further research is warranted to delineate the immune pathways controlling viral replication.

## Data Availability

The raw data supporting the conclusions of this article will be made available by the authors, without undue reservation.

## References

[1] Liu J Liang W Jing W Liu M. Countdown to 2030:eliminating hepatitis B disease, China. *Bull World Health Organ.* (2019) 97:230–8. 30992636 10.2471/BLT.18.219469 PMC6453311

[2] Mason WS Aldrich C Summers J Taylor JM. Asymmetric replication of duck hepatitis B virus DNA in liver cells: Free minus strand DNA. *Proc Natl Acad Sci U S A.* (1982) 79:3997–4001. 6287459 10.1073/pnas.79.13.3997 PMC346563

[3] Wu TT Coates L Aldrich CE Summers J Mason WS. In hepatocytes infected with duck hepatitis B virus, the template for viral RNA synthesis is amplified by an intracellular pathway. *Virology.* (1990) 175:255–61. 2155510 10.1016/0042-6822(90)90206-7

[4] Zoulim F. New insight on hepatitis B virus persistence from the study of intrahepatic viral cccDNA. *J Hepatol.* (2005) 42:302–8. 15710212 10.1016/j.jhep.2004.12.015

[5] Dandri M Burda MR Will H Petersen J. Increased hepatocyte turnover and inhibition of woodchuck hepatitis B virus replication by adefovir in vitro do not lead to reduction of the closed circular DNA. *Hepatology.* (2000) 32:139–46. 10869302 10.1053/jhep.2000.8701

[6] Moraleda G Saputelli J Aldrich CE Averett D Condreay L Mason WS. Lack of effect of antiviral therapy in nondividing hepatocyte cultures on the closed circular DNA of woodchuck hepatitis virus. *J Virol.* (1997) 71:9392–9. 9371599 10.1128/jvi.71.12.9392-9399.1997 PMC230243

[7] Xing TJ Zhao KY Li WT Wang LJ Lu FM. [Association between HBV viral load and severity of liver inflammation in patients with chronic hepatitis B virus infection]. *Zhonghua Gan Zang Bing Za Zhi.* (2023) 31:954–60. 10.3760/cma.j.cn501113-20230820-00061 37872091 PMC12850652

[8] Loomba R Liang TJ. Hepatitis B reactivation associated with immune suppressive and biological modifier therapies: Current concepts, management strategies, and future directions. *Gastroenterology.* (2017) 152:1297–309. 28219691 10.1053/j.gastro.2017.02.009 PMC5501983

[9] Revill PA Chisari FV Block JM Dandri M Gehring AJ Guo H A global scientific strategy to cure hepatitis B. *Lancet Gastroenterol Hepatol.* (2019) 4:545–58. 30981686 10.1016/S2468-1253(19)30119-0 PMC6732795

[10] Magri A D’Arienzo V Minisini R Jühling F Wing PAC Rapetti R Inflammatory gene expression associates with hepatitis B virus cccDNA- but not integrant-derived transcripts in HBeAg negative disease. *Viruses.* (2022) 14:1070. 10.3390/v14051070 35632812 PMC9146050

[11] Zhao J Bian D Liao H Wang Y Ren Y Jiang Y Serum HBsAg and HBcrAg is associated with inflammation in HBeAg-positive chronic hepatitis B patients. *Front Cell Infect Microbiol.* (2023) 13:1083912. 10.3389/fcimb.2023.1083912 37065191 PMC10102387

[12] Rockey DC Caldwell SH Goodman ZD Nelson RC Smith AD American Association for the Study of Liver Diseases Liver biopsy. *Hepatology.* (2009) 49:1017–44. 19243014 10.1002/hep.22742

[13] Ishak K Baptista A Bianchi L Callea F De Groote J Gudat F Histological grading and staging of chronic hepatitis. *J Hepatol.* (1995) 22:696–9. 7560864 10.1016/0168-8278(95)80226-6

[14] Knodell RG Ishak KG Black WC Chen TS Craig R Kaplowitz N Formulation and application of a numerical scoring system for assessing histological activity in asymptomatic chronic active hepatitis. *Hepatology.* (1981) 1:431–5. 7308988 10.1002/hep.1840010511

[15] Volz T Lutgehetmann M Wachtler P Jacob A Quaas A Murray JM Impaired intrahepatic hepatitis B virus productivity contributes to low viremia in most HBeAg-negative patients. *Gastroenterology.* (2007) 133:843–52. 17854594 10.1053/j.gastro.2007.06.057

[16] Levrero M Pollicino T Petersen J Belloni L Raimondo G Dandri M. Control of cccDNA function in hepatitis B virus infection. *J Hepatol.* (2009) 51:581–92. 19616338 10.1016/j.jhep.2009.05.022

[17] Gao Y Li Y Meng Q Zhang Z Zhao P Shang Q Serum hepatitis B virus DNA, RNA, and HBsAg: Which correlated better with intrahepatic covalently closed circular DNA before and after Nucleos(t)ide Analogue Treatment? *J Clin Microbiol.* (2017) 55 2972–82. 28747369 10.1128/JCM.00760-17 PMC5625383

[18] Thompson AJ Nguyen T Iser D Ayres A Jackson K Littlejohn M Serum hepatitis B surface antigen and hepatitis B e antigen titers: Disease phase influences correlation with viral load and intrahepatic hepatitis B virus markers. *Hepatology.* (2010) 51:1933–44. 20512987 10.1002/hep.23571

[19] Li W Zhao J Zou Z Liu Y Li B Sun Y Analysis of hepatitis B virus intrahepatic covalently closed circular DNA and serum viral markers in treatment-naive patients with acute and chronic HBV infection. *PLoS One.* (2014) 9:e89046. 10.1371/journal.pone.0089046 24551214 PMC3923869

[20] Wang Q Luan W Warren L Fiel MI Blank S Kadri H Serum hepatitis B surface antigen correlates with tissue covalently closed circular DNA in patients with hepatitis -associated hepatocellular carcinoma. *J Med Virol.* (2016) 88:244–51. 26174662 10.1002/jmv.24326

[21] Cheng PN Liu WC Tsai HW Wu IC Chang TT Young KC Association of intrahepatic cccDNA reduction with the improvement of liver histology in chronic hepatitis B patients receiving oral antiviral agents. *J Med Virol.* (2011) 83:602–7. 21328373 10.1002/jmv.22014

[22] Lin LY Wong VW Zhou HJ Chan HY Gui HL Guo SM Relationship between serum hepatitis B virus DNA and surface antigen with covalently closed circular DNA in HBeAg-negative patients. *J Med Virol* (2010) 82:1494–500. 20648602 10.1002/jmv.21863

[23] Guner R Karahocagil M Buyukberber M Kandemir O Ural O Usluer G Correlation between intrahepatic hepatitis B virus cccDNA levels and other activity markers in patients with HBeAg-negative chronic hepatitis B infection. *Eur J Gastroenterol Hepatol.* (2011) 23:1185–91. 21934508 10.1097/MEG.0b013e32834ba13a

[24] Takkenberg RB Zaaijer HL Menting S Weegink CJ Terpstra V Cornelissen M Detection of hepatitis B virus covalently closed circular DNA in paraffin embedded and cryo-preserved liver biopsies of chronic hepatitis B patients. *Eur J Gastroenterol Hepatol.* (2010) 22:952–60. 20150816 10.1097/MEG.0b013e3283376a63

[25] Larsson SB Eilard A Malmstrom S Hannoun C Dhillon AP Norkrans G HBsAg quantification for identification of liver disease in chronic hepatitis B virus carriers. *Liver Int.* (2014) 34:e238–45. 24118747 10.1111/liv.12345

